# Confined Space Nanoarchitectonics for Dynamic Functions and Molecular Machines

**DOI:** 10.3390/mi15020282

**Published:** 2024-02-17

**Authors:** Katsuhiko Ariga

**Affiliations:** 1 Research Center for Materials Nanoarchitectonics (MANA), National Institute for Materials Science (NIMS), 1-1 Namiki, Tsukuba 305-0044, Japan; ariga.katsuhiko@nims.go.jp; 2 Graduate School of Frontier Sciences, The University of Tokyo, 5-1-5 Kashiwanoha, Kashiwa 277-8561, Japan

**Keywords:** confined space, covalent organic framework, dynamic function, metal–organic framework, molecular machine, nanoarchitectonics, nanospace, surface space

## Abstract

Nanotechnology has advanced the techniques for elucidating phenomena at the atomic, molecular, and nano-level. As a post nanotechnology concept, nanoarchitectonics has emerged to create functional materials from unit structures. Consider the material function when nanoarchitectonics enables the design of materials whose internal structure is controlled at the nanometer level. Material function is determined by two elements. These are the functional unit that forms the core of the function and the environment (matrix) that surrounds it. This review paper discusses the nanoarchitectonics of confined space, which is a field for controlling functional materials and molecular machines. The first few sections introduce some of the various dynamic functions in confined spaces, considering molecular space, materials space, and biospace. In the latter two sections, examples of research on the behavior of molecular machines, such as molecular motors, in confined spaces are discussed. In particular, surface space and internal nanospace are taken up as typical examples of confined space. What these examples show is that not only the central functional unit, but also the surrounding spatial configuration is necessary for higher functional expression. Nanoarchitectonics will play important roles in the architecture of such a total system.

## 1. Introduction

Human development depends on the enrichment of functional materials. As our lives become more diverse, more functions are required. Functional materials must be created to meet these needs. These include generating energy [1,2,3,4,5,6,7], storing energy [8,9,10,11,12,13], detecting and removing environmental hazards [14,15,16,17,18,19], carbon neutrality [20,21,22,23], purifying water [24,25,26,27,28], detecting and handling viruses [29,30,31,32,33], delivering drugs [34,35,36,37,38], treating illness and injury [39,40,41,42,43,44], developing devices for these functions [45,46,47,48,49], and information conversion [50,51,52,53,54]. We must accomplish these targets with scientific development and technological advancements to solve these problems. Here are some existing role models. Various functional expressions, although not exactly the same, can be found in biological systems such as advanced functions in photosynthesis [55,56] and signal transduction systems [57,58]. The advanced functions of living organisms are due to the cleverness of their structural organizations. Many kinds of functional units are rationally arranged to achieve advanced functions. Biological systems have achieved such excellent functional structures in the course of billions of years of evolution. We need to achieve this in a few decades.

**Figure 1:**
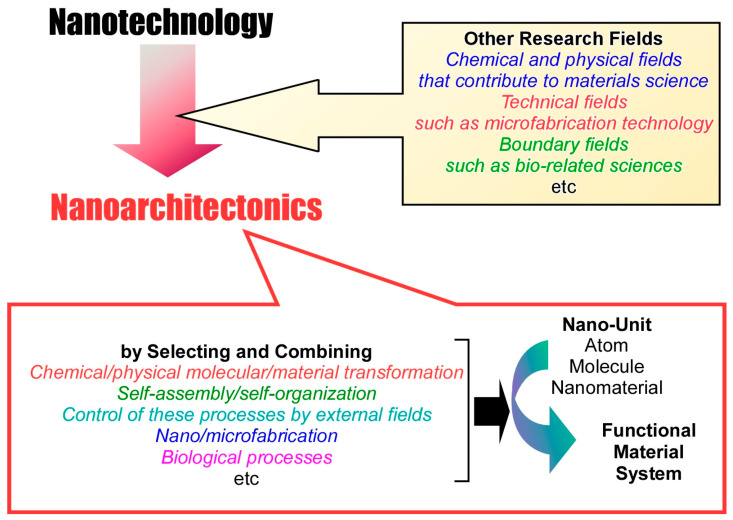
Outline of the post nanotechnology concept nanoarchitectonics, which builds functional material systems from atoms, molecules, and nanomaterials by combining knowledge of nanotechnology with general materials science and other methods.

In order to create artificially precisely structured functional materials, it is necessary, roughly speaking, to develop a system of studies that produces materials, studies that examine unit structures, and studies that create materials from the unit structures. The research field of creating and processing materials has developed rapidly since around the 20th century and is still an active research field. Such fields include organic chemistry [59,60,61,62,63], inorganic chemistry [64,65,66,67,68], polymer chemistry [69,70,71,72,73], coordination chemistry [74,75,76,77,78], supramolecular chemistry [79,80,81,82,83], other material sciences [84,85,86,87,88], and biological sciences [89,90,91,92,93]. Detailed control of fine structure is also being carried out. The breakthrough was the creation of nanotechnology. Nanotechnology has advanced the techniques for elucidating phenomena at the atomic, molecular, and nano-level [94,95,96,97,98]. Since the late 20th century, nanotechnology has flourished. Nanotechnology is said to have been initiated by Richard Feynman in the 20th century [99,100]. As a post nanotechnology concept, nanoarchitectonics has emerged to create functional materials from unit structures (Figure 1) [101]. The first nanoarchitectonics were proposed by Masakazu Aono in the early 21st century [102].

The goal of nanoarchitectonics is to architect functional material systems from atoms, molecules, and nanomaterials by combining knowledge of nanotechnology with general materials science and other methods [103]. Nanoarchitectonics can also be regarded as an integrated concept that combines nanotechnology with other fields of science and technology (chemical and physical fields that contribute to materials science, technical fields such as microfabrication technology, and boundary fields such as bio-related sciences) [104]. Various processes such as manipulation at the atomic/molecular level using nano-units (atoms, molecules, nanomaterials), chemical/physical molecular/material transformation, self-assembly/self-organization, control of these processes by external fields, nano/microfabrication, and biological processes could contribute [105]. The functional materials are constructed by selecting and combining such unit processes. Since multiple processes are often combined in nanoarchitectonics approaches, they are suitable for fabricating complex structures such as hierarchical, asymmetric, and irregular structures [106]. Processes such as self-assembly [107,108,109,110] and template synthesis [111,112,113,114,115] are combined with thin-film processes such as Langmuir–Blodgett (LB) [116,117,118,119,120] and layer-by-layer (LbL) assembly [121,122,123,124,125]. In this case, a hierarchical organization based on a layered structure can be created [126,127,128]. These characteristics are advantages over self-assembly, which is based on simple equilibrium. In addition, the underlying nano-level phenomena include uncertainties such as thermal fluctuations, stochastic distributions, and quantum effects [129]. Therefore, the material architecture is not just a sum, but a harmonization of many effects. The qualities of hierarchy and harmonization are common to the formation of functional structures in biological systems [130]. Therefore, nanoarchitectonics will be a key concept to fill the billions of years of evolution of life with artificial technology in a few decades [131].

The above concepts are general ones that apply regardless of nano-units or materials. Since all matter is originally composed of atoms and molecules, nanoarchitectonics, which assembles matter from atoms and molecules, can be considered a method for the fabrication of all materials. Corresponding to the theory of everything in physics [132], nanoarchitectonics can be considered as a method for everything in material science [133,134]. In fact, the research papers advocating nanoarchitectonics have a very wide range of material processes and applications. In basic fields, nanoarchitectonics can be applied to material synthesis [135,136,137,138,139,140,141], microstructure control [142,143,144,145,146,147,148,149], the elucidation of physical phenomena [150,151,152,153,154,155], and basic life science research [156,157,158,159,160,161]. In the application-oriented fields, there are reports of applications in catalysis [162,163,164,165,166,167], sensors [168,169,170,171,172], devices [173,174,175,176,177,178], energy generation [179,180,181,182,183,184], energy storage [185,186,187,188,189,190], environmental purification [191,192,193,194], drug delivery [195,196,197,198,199,200], and medical-aiming fields [201,202,203,204,205,206].

Consider the material function when nanoarchitectonics enables the design of materials whose internal structure is controlled at the nanometer level. Material function is determined by two elements. These are the functional unit that forms the core of the function and the environment (matrix) that surrounds it. This review article deals with the function when these two elements are constructed with nano-level precision. The functional unit is made by assembling molecules, supramolecules, nanostructures, etc., that have a function. In particular, those with dynamic functions, such as responding to external stimuli or changing morphology, are of particular interest as having more advanced functions [207,208,209,210]. The ultimate example would be a molecular machine in which molecules and supramolecules work like machines [211,212,213,214]. Such a dynamic function would be greatly influenced by the environment around its functional center. By assembling the environment, it will be possible to intentionally perturb the dynamic function. It is also envisioned that functional units incorporated into a regular environment would work in tandem. Conversely, by isolating functional units in a closed-space environment, it will be possible to evaluate the function of a single molecule or unit as an independent basic physical property. In functional material architecture based on nanoarchitectonics, it is important to design and construct not only functional units but also the surrounding space (confined space). Such spaces include environments that are surrounded by nanopore structures, as well as surface spaces that are strongly bounded from the surface. The design of such spaces involves the use of mesoporous materials [215,216,217,218], metal–organic frameworks (MOFs) [219,220,221,222], covalent organic frameworks (COFs) [223,224,225,226], self-assembled monolayers (SAM) [227,228,229,230], Langmuir–Blodgett (LB) film [231,232,233,234], layer-by-layer (LbL) assembly [235,236,237,238], etc.

With the above background, this review paper discusses the nanoarchitectonics of confined space, which is a field for controlling functional materials and molecular machines. The behavior of dynamical functions and molecular machines in such confined spaces will be discussed by looking at recent examples. The first few sections will introduce some of the various dynamic functions in confined spaces, considering molecular space, materials space, and biospace in terms of their size and morphology. In the latter two sections, examples of research on the behavior of molecular machines, such as molecular motors, in confined spaces are discussed. In particular, surface space and internal nanospace are taken up as typical examples of confined space. From these examples, it will be demonstrated that in addition to the central nanoarchitectonics of the function of functional unit architecture, the nanoarchitectonics of the confined space that creates its surroundings is important.

## 2. Dynamic Functions in Confined Space

Of course, molecules and materials have various properties. Functions are often expressed in response to external stimuli. The process involves dynamic changes in properties, such as certain motions and changes in optoelectronic properties. The form of functional expression differs greatly when the phenomenon occurs in open space and when it occurs in confined space. Especially, dynamic functions are strongly perturbed by the confined space. This is not only the case for dynamic functions of sophisticated molecules such as in molecular machines, but also for various other conventional materials functions. The following sections will illustrate some of the behaviors of various functions in confined space, classifying confined space into molecular-level space and material-level space. Another section also summarizes the functions in the space formed from biomolecules as a well-designed space.

### 2.1. Molecular Space

Various molecules are susceptible to perturbation within a space of similar size to that of the molecule. Such molecular spaces include caged molecules, so-called host molecules such as cyclodextrins [239,240,241,242,243] and crown ethers [244,245,246,247,248], and supramolecularly formed spaces [249,250,251,252,253,254]. Some examples of studies on the properties of various molecules in those spaces are given below.

**Figure 2:**
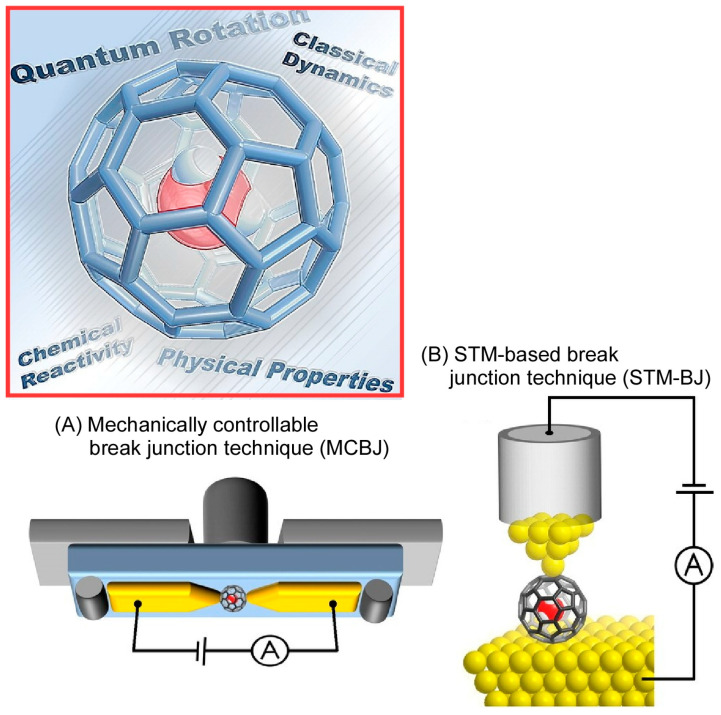
Investigation on a water molecule anchored in the C60 fullerene (H_2_O@C_60_): (**A**) mechanically controllable break junction technique (MCBJ); (**B**) STM-based break junction (STM-BJ). Reprinted with permission from Reference [255]. Copyright 2023 Oxford University Press.

Hashikawa and Murata discuss in a recent review the C_60_ fullerene with a single water molecule [255], The water molecule anchored in the C_60_ fullerene (H_2_O@C_60_) is the ultimate form of water obtained by three-dimensional fractionation. Anomalous behaviors of water molecules without hydrogen bonding have been discovered one after another. Water confined in the sub-nanospace formed by carbon nanomaterials may exhibit fascinating properties that cannot be observed in the bulk environment. The mechanically controllable break junction technique (MCBJ) was applied under ultra-high vacuum conditions of 300 K (Figure 2A). In both junctions (with and without H_2_O in the C_60_ molecule), the electronic conductance of a single molecule was 0.25 ± 0.05 G0 (G0: conductance quantum). This means that the encapsulated H_2_O molecule was almost completely isolated from the external electronic field. Under room temperature conditions, a more sensitive method (STM-based break junction, STM-BJ) using a scanning tunneling microscope (STM) was applied (Figure 2B). The conductance of H_2_O@C_60_ incorporating water was only slightly increased relative to C_60_. On the other hand, a considerably larger conductance was observed for Li^+^@C_60_ incorporating lithium ions. The energy levels of the conduction orbitals are altered by the encapsulation of H_2_O (increase) and Li^+^ (decrease). The single molecule conductance of C_60_ is modulated precisely by the doped internal entity.

**Figure 3:**
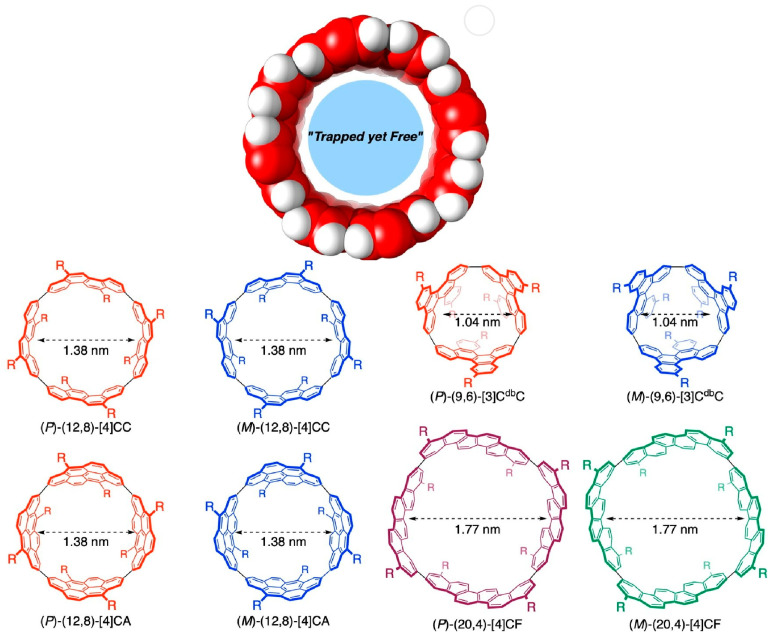
Molecular nanospace of discrete molecular peapods for supramolecular complexion. Reprinted with permission from Reference [259]. Copyright 2023 Oxford University Press.

The supramolecular complexes of carbon nanotubes and trapped fullerenes are called peapods [256,257,258]. Matsuno and Isobe discuss peapod complexes that incorporate molecules in an annular closed space (Figure 3) [259]. By trapping many molecules in such cylindrical nanospaces, a variety of peapod structures are nanoarchitectonized. Especially, atomically precise structures can be created using molecular segments of carbon nanotubes. Supramolecular chemistry of discrete molecular peapods is being investigated by such nanoarchitectonic designs. Peapods are assembled by weak intermolecular interactions such as van der Waals interactions and CH-π hydrogen bonds. Because of the presence of multiple interactions, the aggregation forces are strong enough to result in surprisingly high aggregation constants in solution. The multiple interactions allow for dynamic motion of the trapped guest both in the solution phase and in the solid state. The latter dynamic motion of molecular peapods in the solid state is an interesting fact. This motion is particularly unusual and can occur at very high rotational frequencies in the inertial regime.

**Figure 4:**
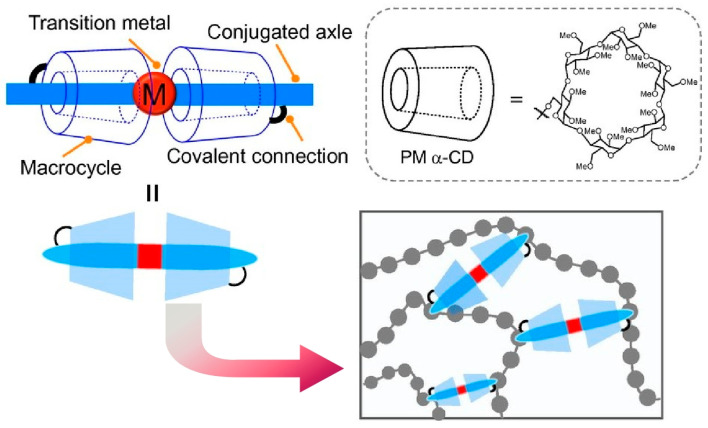
Entrapment of transition metals into a protective environment using permethylated α-cyclodextrin-based macrocyclic compounds. Reprinted with permission from Reference [260]. Copyright 2023 Oxford University Press.

Confining transition metal complexes in molecular space allows one to control their excited state dynamics and chemical reactivity. Masai incorporated transition metals into a protective environment using permethylated α-cyclodextrin-based macrocyclic compounds (Figure 4) [260]. Focusing on the characteristics of the incorporated platinum acetylide complexes, their unique chemical reactivity and optical properties are discussed. The protection of transition metal complexes into the molecular space prevents undesirable conformational changes and chemical reactions and often enhances their functional properties. The stability of transition metal complexes can be precisely controlled by guiding the desired electronic pathways and chemical reactions. Macrocyclic compounds shield excited species from thermal fluctuations in solution and intermolecular interactions in the solid state. Thereby, luminescence efficiency is improved by suppressing undesirable pathways. The molecular space created by the macrocyclic compound dynamically stabilizes the transition metal center against other chemical reagents and electrons via steric hindrance. It will be useful not only for platinum acetylide complexes but also for various transition metals and ligands, allowing precise control of the dynamics and reaction pathways of various transition metal complexes. After kinetically stabilizing the reaction centers, novel reactivity can be radically explored. Through such nanoarchitectonic design, transition metal complexes with novel reactivity can be developed. For example, it would possibly lead to the development of biomimetic sensors and solid-state light-emitting materials.

**Figure 5:**
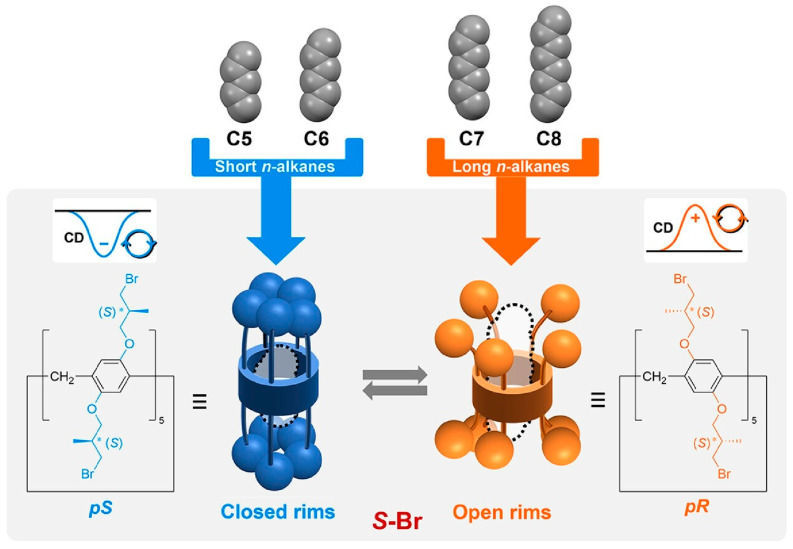
Nanospaces of the chiral substituted pillar[5]arene for the discrimination of n-alkanes with different lengths: short alkane inclusions with a closed configuration in the pS form and longer alkane accommodation with open configuration in the pR form. Reprinted with permission from Reference [261]. Copyright 2023 American Chemical Society.

Some host molecules can complex with alkanes through hydrophobic cavities. Size selectivity can distinguish between the bulkiness of linear, branched, and cyclic alkanes. However, distinguishing the length of n-alkanes remains a difficult task. In particular, there are few examples of the length recognition of linear alkanes using optical aspects such as chirality. The chirality of a well-designed host molecule can be reversed by the guest molecule entering the host cavity. However, n-alkanes are neutral, achiral, linear molecules, making specific interactions difficult to induce. Fa, Ogoshi, and co-workers have demonstrated systems that exhibit different behavior with different lengths of n in n-alkanes (Figure 5) [261]. The nanospaces of the chiral substituted pillar[5]arene are electron-rich and can accommodate n-alkanes. The planar chiral isomers are sensitively inverted depending on the length of the complexed n-alkane. In short alkane inclusions such as n-pentane and n-hexane, the chiral substituted pillar[5]arene complexes assume a closed configuration. In longer n-alkanes, they can take an open configuration. The inclusion of short n-alkanes such as n-pentane tended to shift the chiral substituted pillar[5]arene to the pS form. On the other hand, when long n-alkanes such as n-heptane were included, they tended toward the pR form. Thus, the planar chirality of the chiral-substituted pillar[5]arenes converted in response to slightly varying length n-alkanes, and the CD spectra showed different Cotton effects. The differences in isomeric stability that led to these results were also evaluated by crystal structure and theoretical calculations. Furthermore, as the temperature was varied, the diastereomeric ratio also changed with the inversion of the planar chirality. For n-hexane, an intermediate length n-alkane, the pR form of the chiral substituted pillar[5]arene was dominant at high temperatures, whereas the pS form was dominant at low temperatures. This nanoarchitectonics using the molecular space of the chiral-substituted pillar[5]arene provides an example of how a chiral substrate can change its conformation in response to slight differences in an artificial supramolecular system. This can be thought of as analogous to fine molecular discrimination in natural systems.

**Figure 6:**
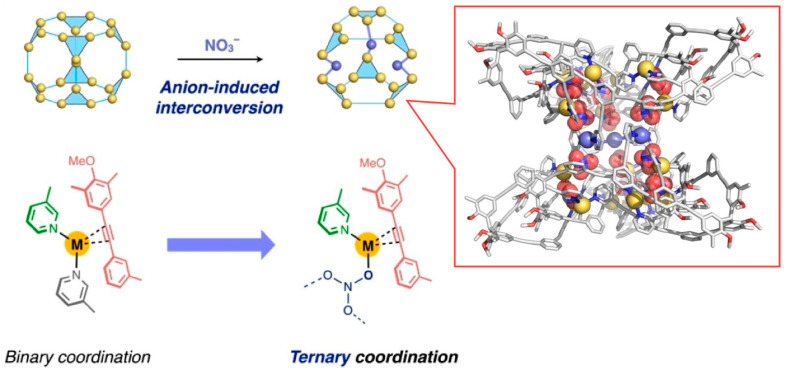
Molecular spaces using interconversion triggered by anion exchange of entangled (Ag_3_L_2_)n polyhedra with conventional metal–pyridyl coordination and relatively weak metal–acetylene interactions. Reprinted with permission from Reference [264]. Copyright 2023 Wiley-VCH.

One of promising nanoarchitectonic methods for creating molecular nanospaces with precise structure is the fabrication of molecular capsules [262,263]. This is a molecular confined space formed by creating a defined coordination structure of metal ions and organic ligands. A variety of molecular spaces can be created by the combination of metal ions and ligands. Domoto et al. reported the creation of molecular spaces using interconversion triggered by the anion exchange of entangled (Ag_3_L_2_)_n_ polyhedra (Figure 6) [264]. The contribution of metal–acetylene interactions yields concave polyhedra in which the metal is inserted into the main framework. The entangled (M_3_L_2_)_n_ polyhedral complexes are stabilized by conventional metal–pyridyl coordination and relatively weak metal–acetylene interactions. Counteranion exchange of these complexes with nitric acid (NO_3_^−^) ions results in metal insertion between the metal centers. A heterogeneous ternary coordination scheme of acetylene, pyridyl, and nitric acid donor is then formed on the metal center. This results in a localized cleavage of the highly entangled three-branched topology and the creation of an extended and complex three-dimensional structural framework. This transformation is accompanied by a change in the cooperative coordination mode from a binary mode to a ternary mode. Heteroleptic cooperative coordination incorporating two or more coordination elements is a useful strategy for achieving molecular complexity in nanostructures and molecular space. Furthermore, it is expected to lead to the construction of nanomaterials with a higher-order molecular order and their precise structural manipulation.

**Figure 7:**
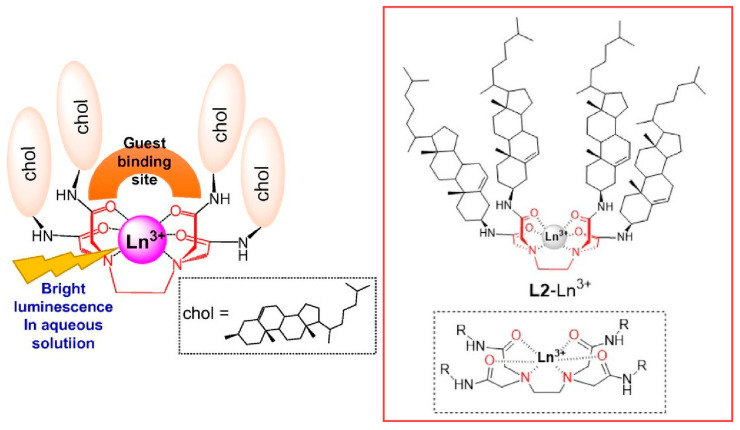
A hexadentate chelate ligand based on ethylenediaminetetraacetic acid with four choles-teryl groups as a molecular space material for stable 1:1 complexes with lanthanide ions exhibiting amphiphilic properties and long-lived emission. Reprinted with permission from Reference [265]. Copyright 2023 Oxford University Press.

Controlling the properties of metal members immobilized in the molecular space has also been well explored. Mieda, Shinoda, and co-workers synthesized a hexadentate chelate ligand based on ethylenediaminetetraacetic acid with four cholesteryl groups as a molecular space material (Figure 7) [265]. This hexadentate chelate ligand forms stable 1:1 complexes with lanthanide ions. The resulting L2-lanthanide complex formed stable self-assemblies with an average particle size of about 50 nm in ethanol solution. The hexadentate chelate lanthanide complex showed amphiphilic properties and long-lived emission. When sodium 2-naphthoate was added as a guest anion, it formed a 1:2 complex with the lanthanide complex. The sensitized luminescence intensity of the complex is enhanced because the large vacancy site of the hexadentate chelate ligand allows the guest molecule to coordinate near the metal center. When 4-alkylbenzoates were used as guest anions, the intensity of sensitized luminescence was strongly dependent on the length of the alkyl chains of the guest molecules. A marked enhancement of luminescence was observed for the most hydrophobic of these guest molecules. Exciton-bound circular dichroism spectra were also obtained. This Cotton effect is due to the chirality of the cholesteryl moiety in the host lanthanide complex. Lanthanide complex nanoarchitectonics using this molecular space could be a platform for nanomaterials that emit light in aqueous solution.

**Figure 8:**
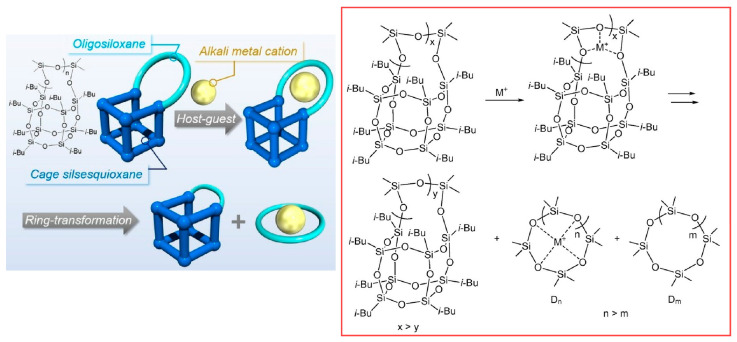
Cyclic compounds by combining cage silsesquioxane with oligo(dimethylsiloxane) as hosts for alkali metal cations with ring transformation behaviors depending on the ring size. Reprinted with permission from Reference [266]. Copyright 2023 Oxford University Press.

The dynamic function of the binding of metal ions to molecular spaces and the subsequent conformational changes have also been investigated. Okamoto et al. nanoarchitectonized cyclic compounds by combining cage silsesquioxane with oligo(dimethylsiloxane) (Figure 8) [266]. Specifically, side-chain ring-opened cagesilsesquioxane was fused with oligo(dimethylsiloxane) to synthesize cyclic molecules based on an inorganic backbone. The nanoarchitectonically synthesized cyclic compounds served as hosts for alkali metal cations (Li^+^, Na^+^, K^+^). Furthermore, in the presence of guest cations, ring transformation was observed to occur depending on the ring size. In addition, other host molecules, including cagesilsesquioxane, and counter anions that can limit ring transformation are being investigated.

Molecular spaces range from those in which the molecules themselves, such as fullerenes, form a space, to those in which the supramolecular structure constitutes a space. The size of the space is at the molecular level, and the functions of the molecules and ions trapped in it are dynamically perturbed. It is also possible to trap molecules such as water, and research development in the field of basic physical chemistry is also expected.

### 2.2. Materials Space

For dynamic functional control by confined space at the material level, materials that encapsulate regular nanospaces are useful. For example, various mesoporous materials [267,268,269,270], layered compounds [271,272,273,274], metal–organic frameworks (MOFs) [275,276,277,278], covalent organic frameworks (COFs) [279,280,281,282], and related structures [283,284,285,286] are used to provide nanospaces in material systems. Some recent examples of such research are discussed below.

**Figure 9:**
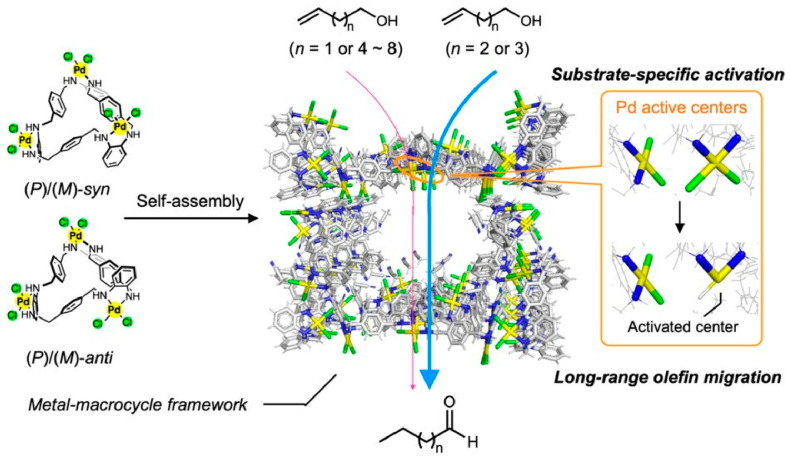
The substrate-specific long-range olefin transfer reaction of alkenyl alcohols catalyzed by a metal-macrocycle framework, a porous supramolecular crystal, as a dynamic functional control in the material space. Reprinted with permission from Reference [287]. Copyright 2023 Oxford University Press.

Tashiro, Ehara, Shionoya, and coworkers report the substrate-specific long-range olefin transfer reaction of alkenyl alcohols catalyzed by a metal–macrocycle framework, a porous supramolecular crystal, as a dynamic functional control in the material space (Figure 9) [287]. The palladium centers precisely arranged in the metal–macrocycle framework are chemically activated by alkenyl alcohols of a certain chain length. In substrate-specific long-range olefin transfer reactions, the metal–macrocycle framework acts as a heterogeneous catalyst that discriminates slight differences in chemical structure, such as chain length or branching structure, of the substrate. Substrate-specific conversion of alkenyl alcohols to aldehydes or ketones is made possible by the metal–macrocycle framework in catalytic amounts. Even slight differences in the framework structure of the reacting substrates can be rigorously identified by the catalyst. For example, aromatic olefins, which normally do not react with non-activated metal–macrocycle frameworks, were converted to olefin migration products at high conversion rates even when reactive substrates were used as additives. The degree of activation can be greatly modulated by various chemical structure features of the additive (hydroxy groups, carboxy groups, double bonds, and specific chain lengths). Activation of organometallic catalytic centers is generally achieved by the removal of the coordinating solvent and ligands by heat treatment or other means. On the other hand, few metal catalytic centers are controlled by the substrate as in the above examples. This means that the activation of the metal catalytic center is regulated by the substrate, product, and coenzyme of the reaction, as is the case with enzymes. This could be a nanoarchitectonics design guideline to give organometallic catalysts high substrate specificity like natural enzymes.

**Figure 10:**
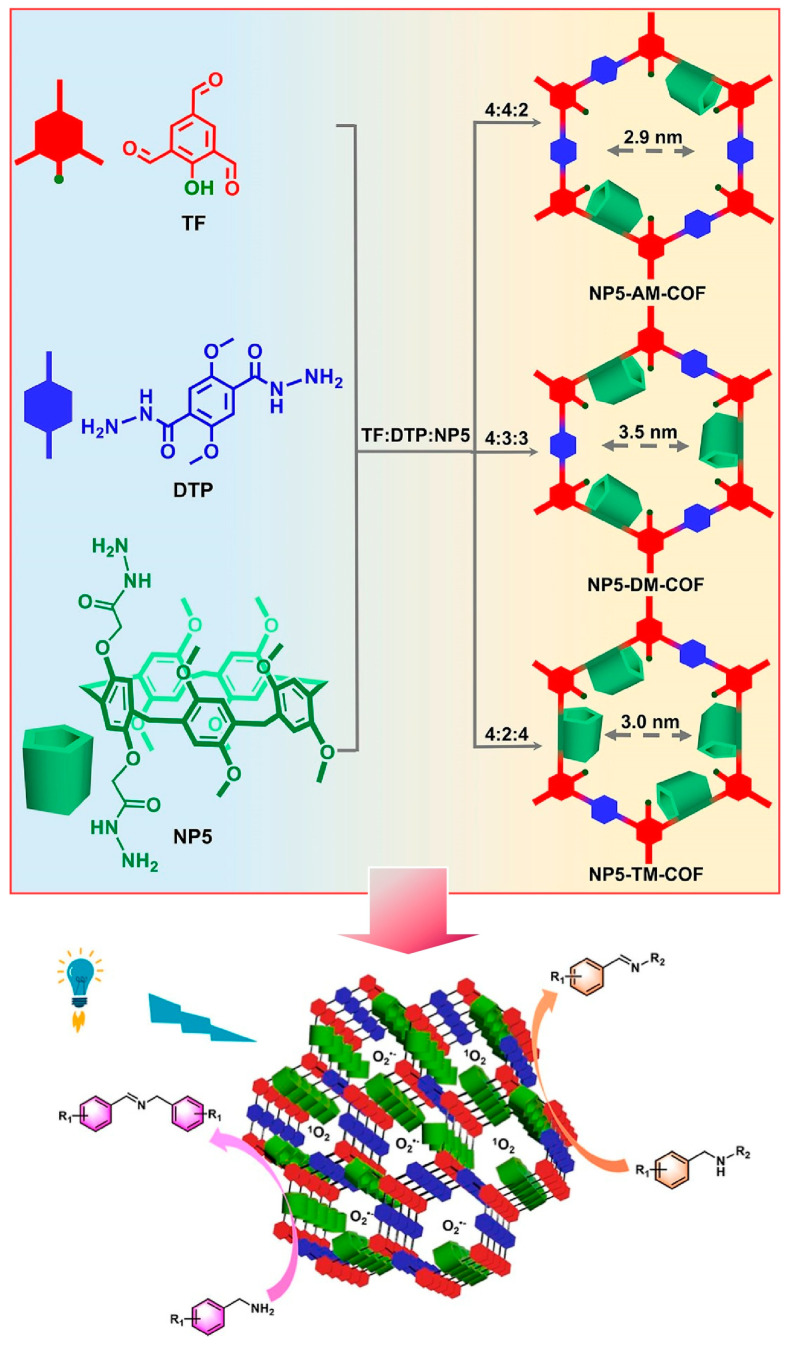
A rigid ring structure, pillar allenes, in a network of COFs with advanced photocatalytic functionality. Reprinted with permission from Reference [288]. Copyright 2022 American Chemical Society.

COFs are crystalline porous materials with tailor-made functionalities. However, photocatalytic functionality in COFs leaves room for development due to the relatively limited choice of constituent elements. Yang and co-workers have created a rigid ring structure in a network of COFs with a nanoarchitectonics approach incorporating pillar allenes. The formed rigid ring structures in a network of COFs produced advanced photocatalytic functionality (Figure 10) [288]. By varying the content of pillararenes, which provide electron-rich cavities, a confined molecular space for exciton transfer and carrier transport was generated. In addition, new interfaces can be created to interact with the photogenerated charge carriers. These material designs produce catalysts that facilitate the efficient oxidation of amines to imines. Specifically, the COF is fabricated by the co-condensation of 1,3,5-tricarbaldehyde, 2,5-dimethoxy terephthalohydrazide, and functionalized pillar[5]arenes. To create COFs with different photophysical and photochemical performances, a nanoarchitectonics strategy was used to incorporate different amounts of methoxy groups into the pore walls. The presence of methoxy groups stabilizes interlayer interactions by weakening the polarization of C-N bonds. This allows the crystallinity of the framework to be tuned by the appropriate crystallinity and the presence of pillar[5]arenes. The separation and transfer of photogenerated electron holes is also regulated. The photocatalytic activity is controlled by the amount of pillar[5]arene present in the linker portion, resulting in different imine conversion capacities. The structural factors of reversible dynamic covalent bonding, interlayer interactions, and electron-rich pillar arenes control the ability of the active site to separate and transport photogenerated electrons. The COF of the pillar arene framework as well as various photocatalytic nanoarchitectonics design hints. For example, it will also facilitate the development of green-light-responsive COF catalysts.

**Figure 11:**
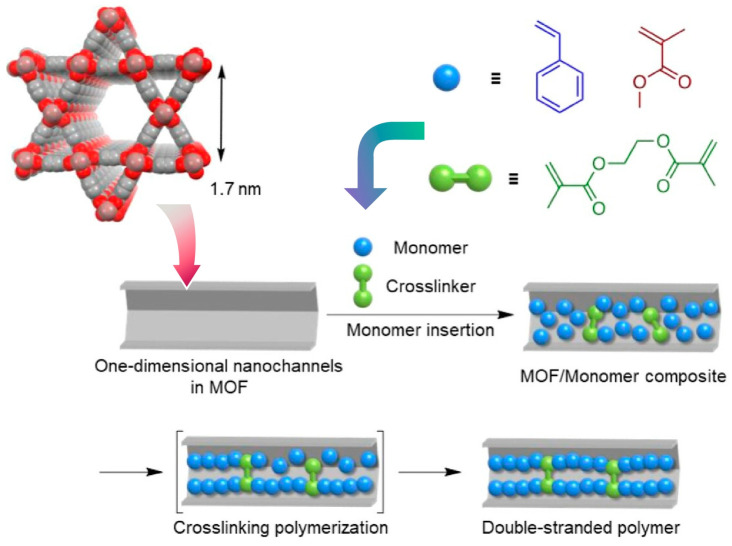
Synthesis of double-stranded polymers such as polystyrene and polymethyl methacrylate in the MOF with one-dimensional channels with diameters similar to the thickness of the two polymer chains. Reprinted with permission from Reference [289]. Copyright 2023 American Chemical Society.

One-dimensional material nanospaces are used to fabricate defined polymer structures. For example, isolated double-stranded polymers are nanoarchitectonized in material nanospace. Such double-stranded structures are common in biopolymers. However, a general and versatile methodology for making double-stranded synthetic polymers has not yet been developed. Uemura and co-workers proposed a new nanoarchitectonics method to synthesize double-stranded polymers such as polystyrene and polymethyl methacrylate (Figure 11) [289]. In the proposed method, crosslinking radical polymerization was carried out in the pores of a MOF with one-dimensional channels with diameters similar to the thickness of the two polymer chains. First, vinyl monomers were encapsulated and polymerized together with a cross-linking agent within the nanochannels of a one-dimensional MOF. As a result, two chains of vinyl polymer were formed within the one-dimensional nanospace. The conformation of the two polymers evolves by being effectively constrained within the pores of the one-dimensional MOF. The cross-linking reaction between the two polymer chains is highly controlled. Undesirable cross-linking between polymer chains in different pores is also prevented. This methodology has been demonstrated with common vinyl polymers such as polystyrene and polymethyl methacrylate. This guarantees the versatility of the technique. As a further feature, the resulting double-stranded polymers were soluble in many organic solvents even at high cross-linking rates, unlike conventional cross-linked polymers. Extension to MOF of different sizes and shapes will contribute to new polymer nanoarchitectonics, such as ladder polymers and polymer bundles consisting of only a specific number of polymers. The development of a series of bundle topological polymers would also be possible.

**Figure 12:**
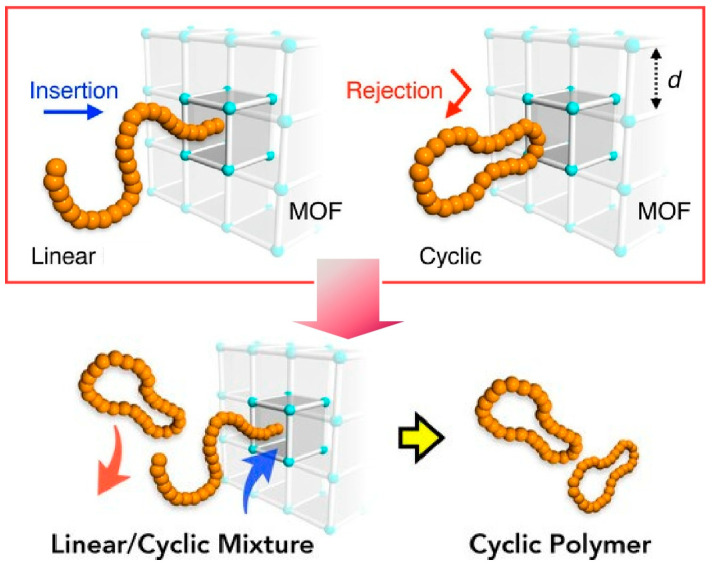
Efficiently separation of cyclic poly(ethylene glycol) from a chaotic mixture containing linear impurities at MOF with regular one-dimensional nanochannels with a precise aperture size. Reprinted with permission from Reference [290]. Copyright 2021 Wiley-VCH.

Some studies have attempted to use the nanopores of the MOF not as a synthesis site for macromolecules but as a space for macromolecular sorting. Hosono, Uemura, and co-workers demonstrated a new macromolecular separation technique that can practically and efficiently separate cyclic poly(ethylene glycol) from a chaotic mixture containing linear impurities on a gram basis (Figure 12) [290]. The separation medium, MOF, was [Zn_2_(1,4-ndc)_2_ted]n (ndc = naphthanlenedicarboxylate, ted = triethylenediamine). This MOF has regular one-dimensional nanochannels with an aperture size of d = 5.7 Å in the c-axis direction. Creating a column packed with MOFs with these one-dimensional pores enabled the analysis and preparative chromatographic separation of these topologically different polyethyleneglycols. Gram-scale purification of cyclic polyethylene glycols is also possible by removing linear impurities. These methodologies result in operationally simple purification procedures. Therefore, they are also expected to be applied to further automated multiscale fractionation techniques.

**Figure 13:**
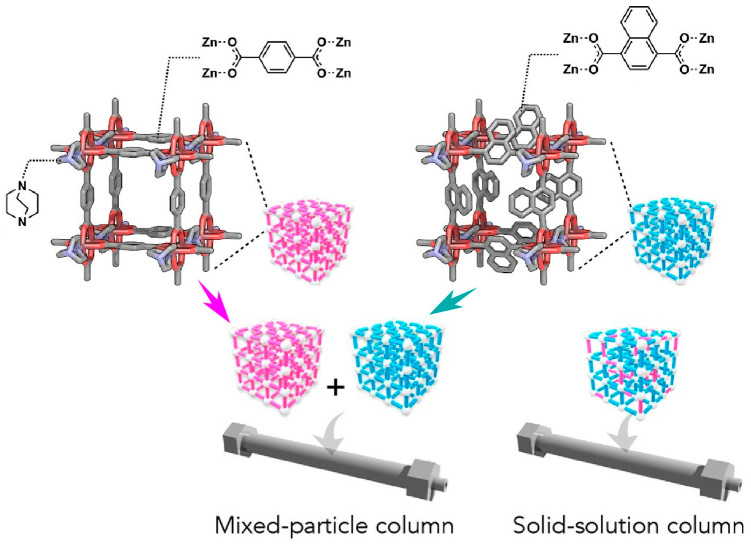
Chromatography stationary phase using mixture (mixed-particle column) and solid solutions (solid solution culms) of multiple MOFs. Reprinted with permission from Reference [291]. Copyright 2023 American Chemical Society.

The nanoarchitectonics of stationary phases in liquid chromatography is of great importance to separation science. As demonstrated by Hosono, Uemura, and coworkers, MOFs with material nanospaces open up new possibilities, such as a chromatography stationary phase creation nanoarchitectonics strategy using solid solutions of multiple MOFs (Figure 13) [291]. In this study, MOFs were prepared using 1,4-benzenedicarboxylate, 1,4-naphthalenedicarboxylate, and 9,10-anthracenedicarboxylate as ligands. Individual MOFs and their mixed particles and solid solutions were prepared in packed columns packed with stationary phases. The retention capacity of polyethylene glycol was investigated by liquid chromatography. The packed columns of MOF packed with binary mixtures of different MOF particles showed a retention capacity that could be estimated from their mixing ratio. On the other hand, columns packed with a mixed linker solid solution MOF showed a large multi-component effect in retention behavior. In some combinations, the mixed-linker solid solution MOF showed a stronger retention than the pure component MOF stationary phase. This specific retention mechanism is attributed to the unique nanostructure formed by the multi-component solid solution MOF, which is thought to be influenced by the balance of two factors: the adsorption interaction and kinetics of the substrates within the MOF pores. The combined effect of both of these countervailing factors will determine the retention of the solid solution column. The mixed MOF approach allows for a myriad of different MOFs and combinations, and it may meet the versatile demands of liquid chromatography stationary phases.

**Figure 14:**
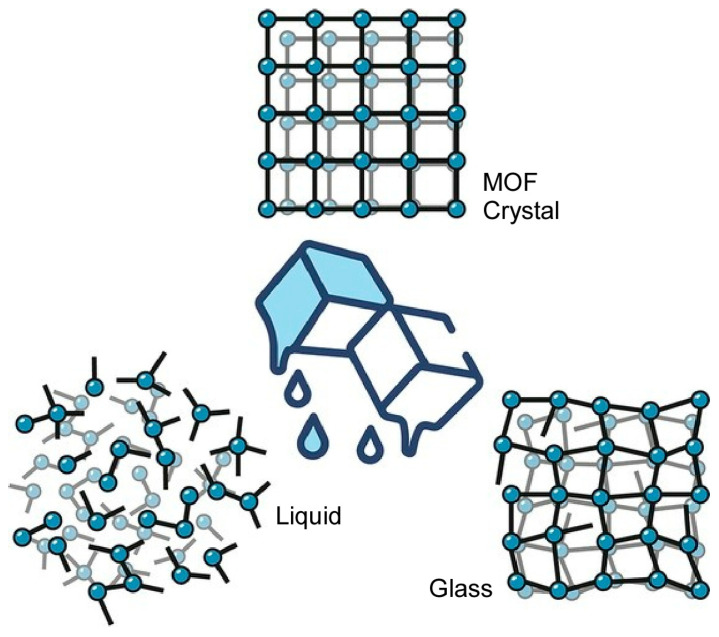
MOFs with disorder such as liquid and glassy state MOFs. Reprinted with permission from Reference [292]. Copyright 2023 Oxford University Press.

In a recent review, Horike discusses the liquid and glassy phases of MOFs (Figure 14) [292]. In many cases, MOFs have been developed on the assumption that they are crystalline. However, the study of systems that include disorder, such as liquid and glassy states, will greatly contribute to the development of functions based on physicochemical properties, such as a high internal degree of freedom, high formability, and softness. In addition to the exploration of basic physical properties, a wide range of material applications such as conductors, membranes, optics, and coatings would attract significant research interest. In one imidazole-based metal–organic framework, the protonated imidazole has a crystal structure in which the protonated imidazole is located between tetrahedrally coordinated Zn^2+^ and an anion chain structure formed by phosphate bridging. When heated to 80 °C, the imidazole exhibits rotational motion. This is the state of the plastic crystal. On further heating, the plastic crystal melts and behaves as a liquid that does not decompose up to a certain temperature. When it is cooled, this melt turns into a glass. Analysis shows that this glass is a network glass consisting of one-dimensional chain-like structures. Understanding the aggregate structure and spatio-temporal behavior of disordered systems such as glasses and liquids is very difficult but also a great challenge. In addition to structural analysis, it is important to explore electronic properties, porosity control, thermal properties, and mechanical properties. In addition, it is also essential to investigate properties that contribute to practical applications, such as stability, safety, and mass synthesis techniques.

This section above has presented several examples of dynamic functions that can be controlled by nanospaces at the material level. Some of the features of functional control are similar to those presented by molecular space. However, material nanospaces are associated with large objects and linked to bulk functions. A typical example would be the material separation function of liquid chromatography with MOFs. The properties of nanospaces in disordered materials remain unexplored, and future research is expected to expand on this topic.

### 2.3. Biospace

Functions in biological systems are often highly efficient and highly selective. Moreover, such functions are achieved under ambient conditions at room temperature and in aqueous environments. These functions are mostly accomplished within specifically designed nanospaces [293,294,295,296]. Mimicking biofunctional structures is significant for developing dynamic functions in confined space. The following sections will present some examples of studies on nanostructures woven by biomolecules and specific structures in the living organisms and their mimics.

**Figure 15:**
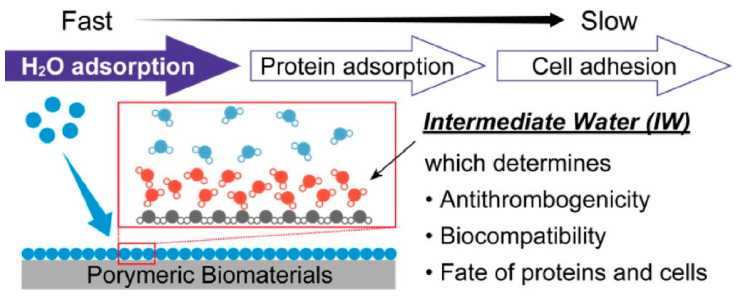
Design of functional biomaterials based on the intermediate water concept as a key parameter in understanding the interactions and functions of diverse biomaterials. Reprinted with permission from Reference [297]. Copyright 2023 Oxford University Press.

Biomaterials such as biopolymers are usually used in water. On their surfaces, hydrated water molecules of varying mobility are formed, including non-frozen water, intermediate water, and free water. Water molecules in the surface space of biomaterials are known to influence biological reactions between biomaterials and biological fluids. In a recent review, Nishimura and Tanaka discussed the design of functional biomaterials based on the intermediate water concept [297]. In particular, they presented their latest results involving synthesis, biological applications, and hydration analysis. The amount of intermediate water is a key parameter in understanding the interactions and functions of diverse biomaterials (Figure 15). Investigations into intermediate water are important not only for basic properties such as biocompatibility, non-staining, and selective adsorption of proteins, but also for applications such as cell adhesion, tissue engineering, and drug delivery systems. Alternatively, it is also useful in the development of multifunctional smart biomaterials for flexible and stretchable electronic devices. The concept of intermediate water has been applied to the development of inorganic biomaterials, as well as organic biomaterials. Investigations into intermediate water will also pave the way for biomedical materials and bio-related devices.

**Figure 16:**
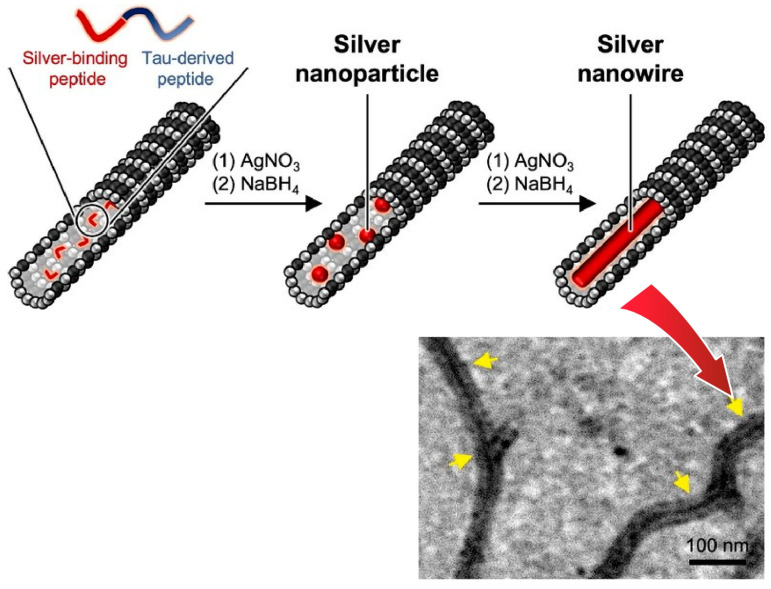
Formation of silver nanoparticles and silver nanowires within the internal space of microtubules of a tandem peptide consisting of a Tau-derived peptide and a silver-binding peptide. Reprinted with permission from Reference [298]. Copyright 2023 Oxford University Press.

Precise biospaces provide a venue for the precise synthesis of inorganic nanomaterials. For example, metal nanoparticles and nanowires are nanomaterials with a wide range of applications and are of great research interest. Using peptides/proteins as templates is a promising strategy for the nanoarchitectonics of homogeneous metallic nanoparticles and nanowires. Inaba, Matsuura, and co-workers reported a tactic to create silver nanoparticles and silver nanowires using the internal space of microtubules of peptide assemblies (Figure 16) [298]. In this technique, silver nanoparticles are grown inside microtubules using a tandem peptide consisting of a Tau-derived peptide and a silver-binding peptide. Of these, silver-binding peptides were isolated by screening phage display libraries against silver nanoparticles. It has the ability to interact with silver clusters and induce the growth of silver nanoparticles. The tandem peptide was incorporated into microtubules and stabilized by cross-linking with glutaraldehyde. By incubating the bionanospace with silver ions and reducing agents, uniform silver nanoparticles were formed in the microtubules. Nanosized materials such as gold nanoparticles, cobalt–platinum nanoparticles, and proteins can also be encapsulated in the microtubules by the nanoarchitectonics method using this bionanospace. The microtubules containing metallic nanostructures constructed in this way have a defined size and morphology. The structural properties are expected to be useful for applications in nanoelectronics and dynamic nanomaterials.

**Figure 17:**
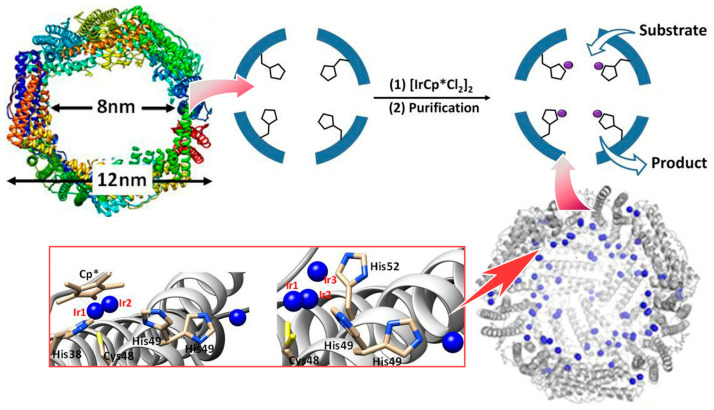
Hybrid bionanocage with an iridium complex immobilized on the internal vacancy of ferritin, a self-assembling protein, as a catalyst for the reduction of substituted acetophenones to the corresponding chiral alcohols with high turnover, free quenching, and high enantioselectivity. Reprinted with permission from Reference [302]. Copyright 2022 Wiley-VCH.

A typical example of high functionality using nanospaces in living organisms is the conversion of substances by enzymes [299,300,301]. In enzymes, there are nanospaces with precisely defined size, shape, and functional groups, called enzyme pockets, which are used to carry out highly efficient and substrate-selective reactions at room temperature. Ueno, Mazumdar, and co-workers have developed a hybrid bionanocage with an iridium complex immobilized on the internal vacancy of ferritin, a self-assembling protein (Figure 17) [302]. The complex composed of organometallic iridium and pentamethylcyclopentadienyl incorporated into the internal cavity of the ferritin cage reduces substituted acetophenones to the corresponding chiral alcohols with high turnover, free quenching, and high enantioselectivity. Iridium-based catalysts are widely known as catalysts for the transfer hydrogenation of carbonyl compounds to the corresponding alcohols. Enantiopure alcohols created by the asymmetric reduction of carbonyl compounds are valuable in the pharmaceutical, flavor, and fragrance industries. In particular, the properties of this bionanospace were modulated by using mutants of ferritin nanocages. Certain mutants of the hybrid bionanocage have increased uptake of iridium complexes and enhanced catalytic activity. The presence of electron-withdrawing substituents increases the apparent rate of reaction (turnover frequency). Based on the size and polarity of the substituted acetophenone in the substrate-binding nanospace, the orientation and binding of the substrate is controlled and the enantioselectivity of the catalytic reaction is tuned. The development of novel mutants of ferritin that selectively incorporate specific guest compounds will likely be extended to applications in a variety of reactions. In the future, it could be applied as a viable catalyst for industrial applications.

**Figure 18:**
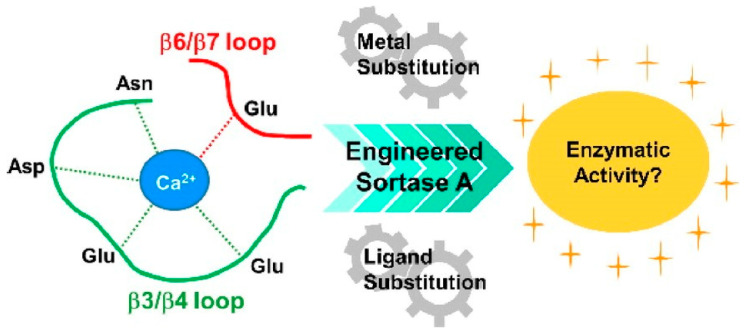
The effects of mutations and various conditions (metal substitution and ligand substitution) on the transpeptidase activity of the enzyme Sortase A. Reprinted with permission from Reference [303]. Copyright 2022 Oxford University Press.

Functional modification of bionanospace by the modulation of proteins and peptides has also been investigated in various ways. For example, the effects of various conditions and mutations on the transpeptidase activity of the enzyme Sortase A were studied by Negi et al. (Figure 18) [303], who examined the effects of alanine mutations in five amino acids involved in Ca^2+^ coordination on enzyme activity. It was found that the effects of amino acids were not equivalent and that the amino acid residues with the strongest influence on enzyme activity were identified. In addition to steric effects, the Lewis basicity of the amino acid side chains and electronic effects are closely related. When Ca^2+^ was examined by replacing it with its homologous elements, Mg^2+^ and Sr^2+^, it was also found that small changes in ionic radius can significantly affect reactivity. Such studies via modulation of the enzyme nanospace are a powerful tool for the study of structure–function relationships. It can also lead to bio-nanoarchitectonics, in which artificial enzymes with new functions can be created by redesigning the coordination site.

**Figure 19:**
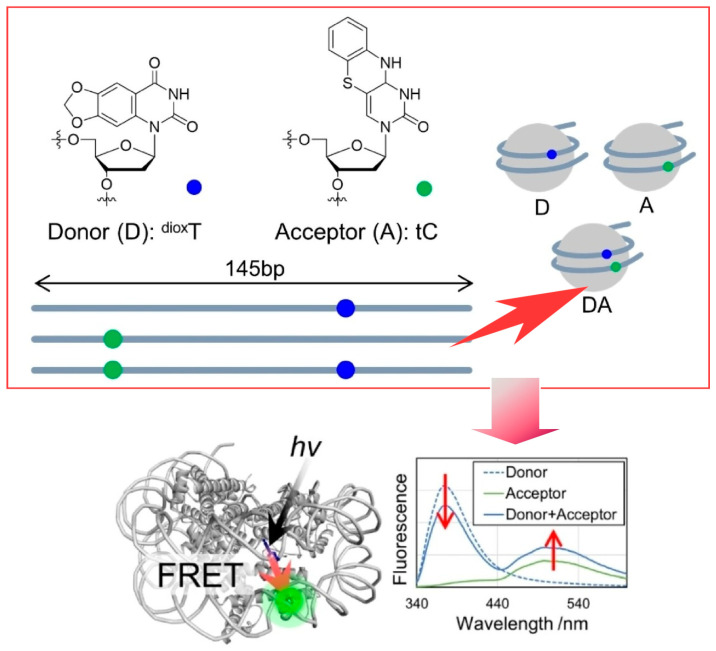
Nucleosomes reconstituted with nucleosomal DNA containing fluorescent nucleosides capable of the Förster resonance energy transfer pair. Reprinted with permission from Reference [304]. Copyright 2023 Wiley-VCH.

Bionanospace can be a site for the precise evaluation of physicochemical phenomena. Precise evaluation of Förster resonance energy transfer can provide insight into the dynamics of biomolecules. For example, the analysis of nucleosomes based on Förster resonance energy transfer is expected to bridge the gap between static structure and dynamic cellular behavior. Hirashima et al. constructed nucleosomes containing nucleobase-Förster resonance energy transfer pairs and used steady-state fluorescence spectroscopy and molecular dynamics simulations (Figure 19) [304]. Nucleosomal DNA containing fluorescent nucleosides that would be the Förster resonance energy transfer pair was synthesized, and modified nucleosomes were reconstituted with it. The Förster resonance energy transfer efficiency was calculated from the analysis of fluorescence spectra. The Förster resonance energy transfer efficiencies of modified nucleosomes with different acceptor positions were compared. Steady-state fluorescence spectrometry of nucleosomes showed different Förster resonance energy transfer efficiencies depending on the donor and acceptor positions. The correlation between the Förster resonance energy transfer efficiency and the position of the Förster resonance energy transfer pair was also verified by molecular dynamics simulations. The Förster resonance energy transfer efficiency pairs used in this approach are located within the helical structure of DNA and can be used without unwanted interactions of fluorophores compared to conventional assays. In the future, the single-molecule Förster resonance energy transfer analysis of nucleosomes is expected.

**Figure 20:**
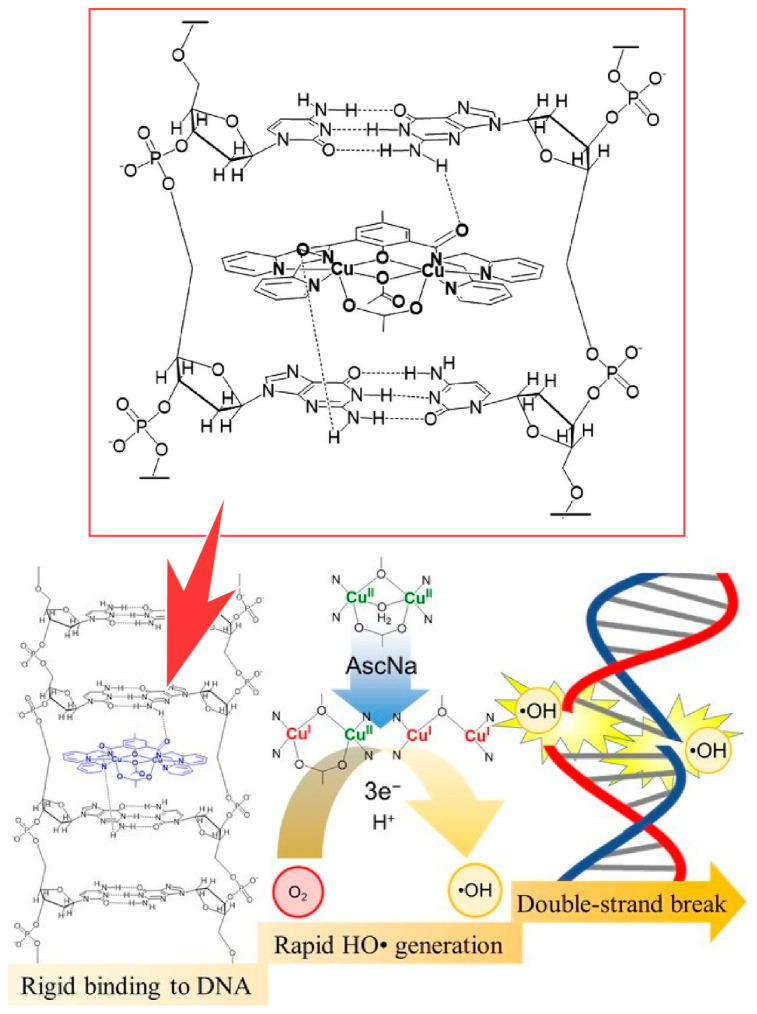
DNA double-strand breaks in bursts via reductive oxygen activation at a di-copper(II) complex with *p*-cresol-2,6-bis(amide ether-dpa) ligands by sodium ascorbate in an air atmosphere. Reprinted with permission from Reference [305]. Copyright 2022 Oxford University Press.

Functional units, such as catalysts, may be incorporated into the bionanospace and express biological-like functions. Kodera and co-workers found that a di-copper(II) complex with *p*-cresol-2,6-bis(amide ether-dpa) ligands can cause bursts of DNA double-strand breaks in an air atmosphere via reductive oxygen activation by ascorbic acid [305]. It was found that the di-copper(II) complex with a 6-bis(amide ether-dpa) ligand causes DNA double-strand breaks in bursts via reductive oxygen activation by sodium ascorbate in an air atmosphere (Figure 20). Spectroscopic, electrochemical, and kinetic studies revealed that the bicopper(II) complex is rapidly reduced by sodium ascorbate to Cu(I)Cu(II) and Cu(I)Cu(I) species. These reduced species are involved in the rate-limiting three-electron reduction of O_2_ to HO^−^, which is responsible for DNA cleavage. Although the di-copper(II) complex is incorporated into the DNA nanospace, the mode of binding to DNA and the rapid HO^−^ formation are key factors that allow the bursting of DNA double-strand breaks. The results obtained provide a new methodology for the development of DNA double-strand break agents and could be developed into useful methods for gene editing and therapeutic applications.

DNA is useful as a method to form nanostructures due to the complementarity caused by specific hydrogen bonds between bases. For example, the creation of specific structures by DNA oligomers is a typical example. Murayama et al. in their recent review discuss the function of acyclic xeno nucleic acids [306]. They envision the potential for the synthesis of artificial genomes and construction of DNA nanostructures and nanomachines. Nanopore sequencing technology for DNA sequencing at the single molecule level is also of interest as an application of nanopore structures to DNA editing. An, Liang, and co-workers have reported on short dsDNA elongation by ligation and PEAR (polymerase-endonuclease amplification reaction) [307]. This method is expected to highlight the high-throughput and accurate detection of mixtures of thousands of short dsDNAs by nanopore sequencing.

Bionanospace can be controlled by various methodologies, such as specific water structures in biomembrane surface space, functional modification through nanospace modulation by peptides/proteins such as modification of enzyme pockets, and the creation of various functional spaces and functional structures by DNA. By modifying biomolecular systems that originally possess such functions and expressing functions through confined space, it is possible to modify functions at a higher level. The examples introduced above are only a small part of the possibilities, and there is a great potential for the development of functions using bionanospace.

## 3. Molecular Machine in Surface Space

Among various dynamic functions, molecular machines in which molecules and supramolecules work like machines are considered to be the ultimate functional systems. In the following two sections, we discuss applications of molecular machines, such as molecular motors, in confined space, where surface space systems, which are three-dimensionally open but bounded by interfacial phenomena, and nanospace systems, which are spatially confined, are considered as fields.

Molecular machine control in the surface space involves a choice of interfaces: liquid interfaces and solid interfaces. A typical example of a liquid interface is the air–water interface. When molecular machines are arranged in a monolayer at the air–water interface, they are constrained to the interfacial space. However, the freedom of motion with respect to the lateral direction is high. In addition, the interface itself can freely expand and compressed. Therefore, the system can be deformed macroscopically in the interfacial lateral direction. While some freedom of molecular motion is maintained, there is no large three-dimensional diffusion, and molecular deformation is constrained to the two-dimensional plane. Taking advantage of this environment, macroscopic interfacial transverse mechanical deformation and nanoscopic molecular changes in the molecular machine can be linked [308]. For example, the workings of a molecular machine can be controlled by large mechanical motions, such as hand movements, i.e., molecular machines can be controlled by simple movements like manual operation [309]. On the other hand, even at solid interfaces, there is freedom of motion in a two-dimensional plane due to surface diffusion and other factors, but the binding of molecular machines is much higher at solid interfaces than at liquid interfaces. Taking advantage of this characteristic, the advanced observation of molecular machines becomes possible [310,311,312]. In a way that is difficult to achieve in solution or at a liquid interface, the morphology of molecular machines can be observed at the molecular (atomic) level of precision at the solid interface. As a result, the rotation of molecular motors can be observed realistically. It is the latter type of molecular machine control at solid surfaces that more strongly reflects the effect of confined space at the nano-level. In the following, I would like to discuss some recent research examples, focusing mainly on molecular machine control at solid interfaces.

For example, unidirectional, repetitive, GHz-frequency rotation can be expected for molecular motors, driven by a fast rotating electric field. The basic behavior must be investigated on a gas-phase solid substrate before it can be applied as a nanostirrer in solution systems. In particular, considerations of factors such as internal charge flow, thermal noise, and molecular flexibility are critical to selecting and predicting the appropriate frequency of the rotating electric field to drive the unidirectional rotation of the molecular motor. Theoretical analysis of two surface-mounted dipole rotors was performed using a combination of quantum mechanical calculations and torque analysis as reported by Zhao, Zhang, Hove, and co-workers [313]. The driving force for the unidirectional rotation of the rotor can be quantified and considered in terms of a torque vector acting on the rotor projected onto the axis of rotation. This torque vector is sensitive to the angle between the dipole arm and the electron field. This is due to the redistribution of atomic charges under the influence of an external electron field. Most of the torques cancel each other out between functional groups, but only the remaining net torque leads to collective intramolecular cooperation. Torque analysis at each rotational step reveals that this promotes unidirectional rotation. Furthermore, for the practical application of a fast-rotating electric-field-driven rotor as a nanostirrer in a fluid, it is necessary to model the rotor assuming a solvent environment under a fast-rotating electric field. It is necessary to determine the shielding of the fast-rotating electric field by the solvent/ion and the effect of the fast-rotating electric field on the orientation of the solvent/ion and its interaction with the rotor molecules.

**Figure 21:**
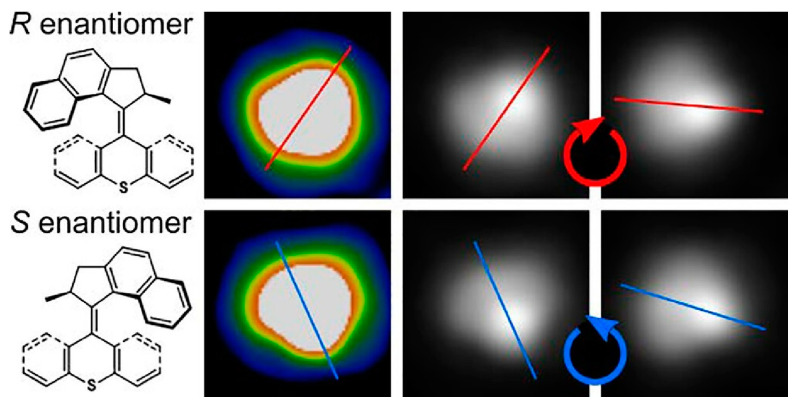
A low-temperature STM observation on the dynamics of a single molecular motor on a Cu(111) surface, where the direction of rotation of independent individual molecules depended on their chiralitys. Reproduced under the terms of the CC-BY license [314]. Copyright 2023 American Chemical Society.

Molecular motors have a chemistry that allows for unidirectional motion. Prezzi, Tour, Grill, and co-workers used a low-temperature scanning tunneling microscope (STM) to study the dynamics of a single molecular motor on a Cu(111) surface (Figure 21) [314]. Dynamics were studied, excited upon excitation by voltage pulses from an STM chip, and rotated around a fixed pivot point. The motor molecules yielded (S) and (R) enantiomers, depending on the result of chemical synthesis. STM observations correlated the dynamics of each molecule with its chirality. The chirality was found to play an important role in the function of the molecular motor. The direction of rotation of independent individual molecules depended on their chirality, which could be determined from STM images. The (S) and (R) enantiomers exhibit different helical structures, (M) and (P). Helix inversion, the thermal relaxation of the helical structure, is an important step in the motor behavior. This occurs in only one direction depending on the chirality. As a consequence of the chiral properties of the motor, different enantiomers of the molecular motor would be expected to rotate in opposite directions. Various results indicate that unidirectional rotation may not be directly related to the motor properties of the molecule. It seems that the molecules are vibrationally excited, resulting in unidirectional rotation in an asymmetric potential energy landscape. Motor structures such as propellers require a higher energy than simply rotating molecules as rigid bodies. Aromatic systems on metals exhibit considerably higher adsorption energies, sticking to the surface and making motor rotation difficult. Correspondingly, they will follow a path with a lower barrier. This is thought to cause rotation in one direction, depending on the chirality of the motor.

**Figure 22:**
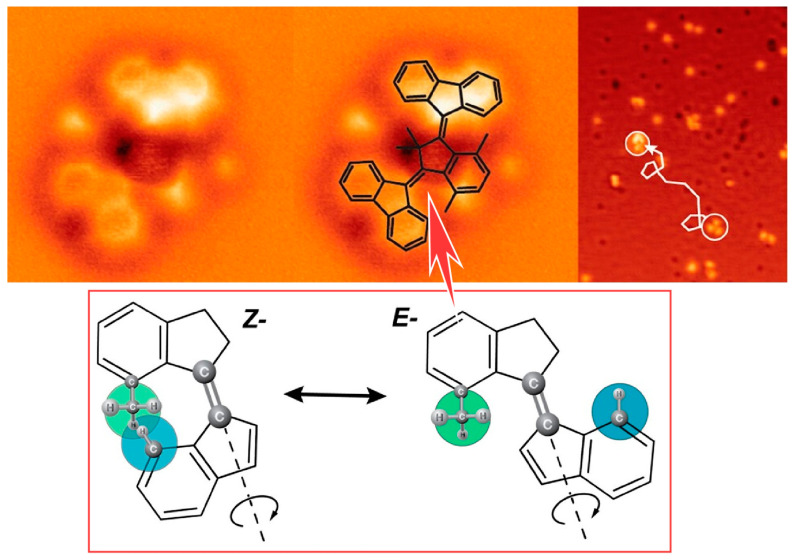
Motion of two-rotor motor molecules with inelastic tunneling electrons on a Cu(111) sur-face in ultrahigh vacuum at 5 K with vibrational excitation to cause switching between different molecular conformations, including conversion of the enantiomeric state of the chiral conformation. Reprinted with permission from Reference [315]. Copyright 2023 American Chemical Society.

The excitation of single molecules by electrons tunneling between the sharp metal tip of the STM and the metal surface can control the dynamics of molecules on the surface. Phenomena such as hopping, rotation, molecular switching, and chemical reactions can be induced by electron tunneling. Feringa, Ernst, and co-workers have investigated the motion of two-rotor motor molecules with inelastic tunneling electrons on a Cu(111) surface in an ultrahigh vacuum at 5 K (Figure 22) [315]. The motor molecules used are based on motors characterized by two sterically overcrowded alkenes. They exist in different helical structures. Thus, the two molecular helical structures generate local asymmetries on both sides of the molecule. Vibrational excitation is observed to cause switching between different molecular conformations, including conversion of the enantiomeric state of the chiral conformation. Vibrational inelastic electron tunneling excitation caused conformational switching, and electronic excitation resulted in E-Z isomerization. This results in the rotation of one of the two rotor units. This process causes the molecules to move across the copper surface.

**Figure 23:**
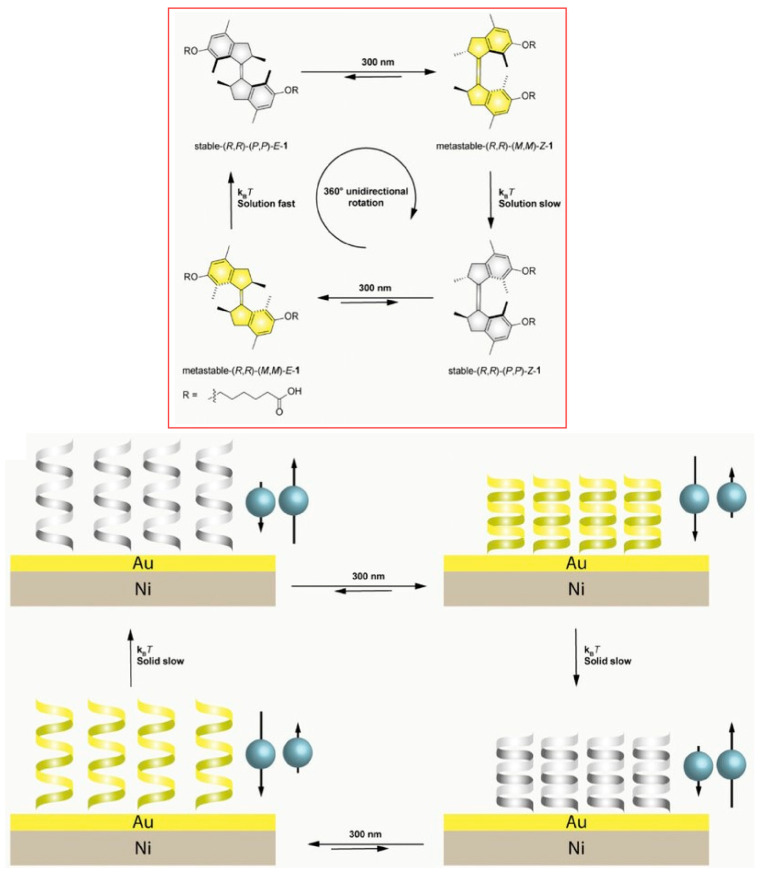
Multi-state spin selectivity in electron transfer through motors based on four different helical configurations switching. Reproduced under the terms of the CC-BY license [316]. Copyright 2021 Wiley-VCH.

The electrons that penetrate a chiral molecule depend on the spin of the electrons. This phenomenon is called chiral-induced spin selectivity. It has been observed in many systems, including chiral molecules, supramolecular structures, polymers, and organometallic thin films. There is potential for applying this phenomenon to molecular motors with controllable chirality and helix states as described above. Cohen, Feringa, Naaman, and co-workers have demonstrated multi-state spin selectivity in electron transfer through motors based on four different helical configurations switching, as measured by magnetoconductive atomic force microscopy (AFM) (Figure 23) [316]. Molecular motors based on sterically overcrowded alkenes exhibit multiple inversions of helical chirality upon photoirradiation and thermal relaxation. Four states with different helical arrangements can be non-invasively interconverted in a specific order. In particular, a surprisingly high spin selectivity compared to the initial structure is observed in the photo-steady-state mixture of isomers obtained by photoisomerization. This opens up the possibility of tuning the spin selectivity on demand with high spatio-temporal precision in a system of molecular motors immobilized in surface nanospaces. These molecular motors can serve as spin filters that can invert the chiral-induced spin selectivity effect at each isomerization step. This could be a guideline for nanoarchitectonics of highly efficient molecular spin filters.

**Figure 24:**
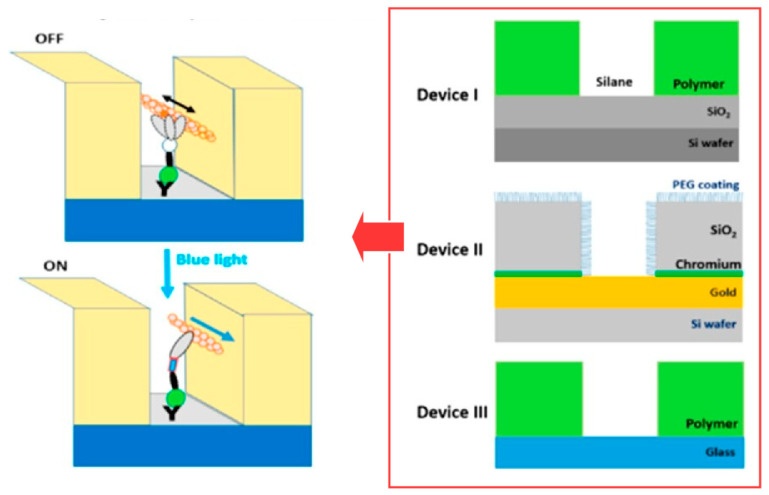
Moving of a biomolecular machine, the artificial myosin motor, in nanochannel space with different surface modifications. Reproduced under the terms of the CC-BY license [321]. Copyright 2023 American Chemical Society.

The best molecular machines are often found in biological systems. They are called biomolecular machines [317,318]. For example, the contractile proteins actin and myosin are very attractive targets for nanotechnology [319,320]. It would be desirable to be able to temporarily turn motor functions on and off in some nanostructured devices. Månsson and co-workers took the tactic of replacing the wild-type myosin II motor fragment with an artificial myosin motor that could be turned on and off locally by changes in illumination [321]. They examined how their biomolecular machine could move in nanochannel space (Figure 24). The artificial motor used can be regarded as a light-sensitive motor. In the absence of blue light, the actin filament powered by the light-sensitive motor moves quite slowly. Conversely, when blue light (470–490 nm) is turned “on”, the actin propulsion velocity increases significantly within seconds. The feasibility of artificial myosin motor motility in nanochannels with different surface modifications was compared. For example, good motility was achieved in Au/SiO_2_-based nanodevices, making the use of photoswitchable motors feasible. However, the reproducibility of the Au/SiO_2_-PEG-based nanodevices was not so good. Further nanospace nanoarchitectonics will help the trapped artificial motors to achieve a high kinetic contrast between “on” and “off” with high motility, which will be useful for effective switching device development.

The design of DNA sequences and the creation of specific complementary pairs can form precise nanostructures. These include objects like molecular machines that move autonomously. In particular, DNA structures that move as if they were walking are called DNA molecular walkers [322,323,324]. Integration of DNA molecular machines such as DNA molecular walkers with DNA origami platforms is a useful approach to the development of advanced nano-robotics with diverse functions. DNA molecular machines provide automated mobility with nanometer resolution. DNA origami technology also provides a sub-microscale platform to guide their locomotion. Wang and co-workers have demonstrated that by driving an advanced light-powered DNA bipedal walker on a rod-shaped DNA origami platform approximately 170 nm in length [325]. Clever use of the fluorescent method allowed the researchers to analyze the self-directed, processual motion of the DNA molecular walker, which, although somewhat more complex on the surface of the DNA oligomers, essentially exhibits the motion of a translational molecular motor. This motion is completely dependent on purely mechanical effects. In other words, if DNA molecular walkers and DNA origami are reasonably optimally matched, functions such as molecular robotics could be achieved. Such a nanoarchitectonics approach would be promising for the development of light-powered DNA nanorobots, automated chemical synthesis by DNA molecular machines, and biomimetic nanomuscles. In particular, light-powered nanomuscles could be powered by rationally designed translational molecular motors. This would serve as the driving element for many nano/micro robots. These could be assembled into even larger functional materials via such DNA nanoarchitectonics.

The immobilization of functional materials on surfaces or their binding to surface nanospaces allows for high-resolution analysis. Such advantages can be recognized in many cases of molecular machines. The details of multi-step or sequential dynamic changes, such as in molecular machines, can be analyzed at high resolution on the surface. This will reveal more detailed mechanisms of molecular machine operation. In addition, dynamic molecular machine motions can become intentional and rational behaviors when coupled with guiding structures such as nanochannels and DNA origami structures. Thus, the immobilization of molecular machines in surface space will pave the way for the advanced analysis of machine behavior and the construction of useful devices.

## 4. Molecular Machine within Nanospace

Material nanospaces can also be envisioned as a binding field for molecular machines. Molecular machines incorporated into molecular assemblies or porous structures are expected to exhibit specific behaviors. In addition, the conjugation of molecular machines and material functions may become possible by confining them in such spaces and endowing them with materials. Below are some examples of studies incorporating molecular machines into nanospaces.

**Figure 25:**
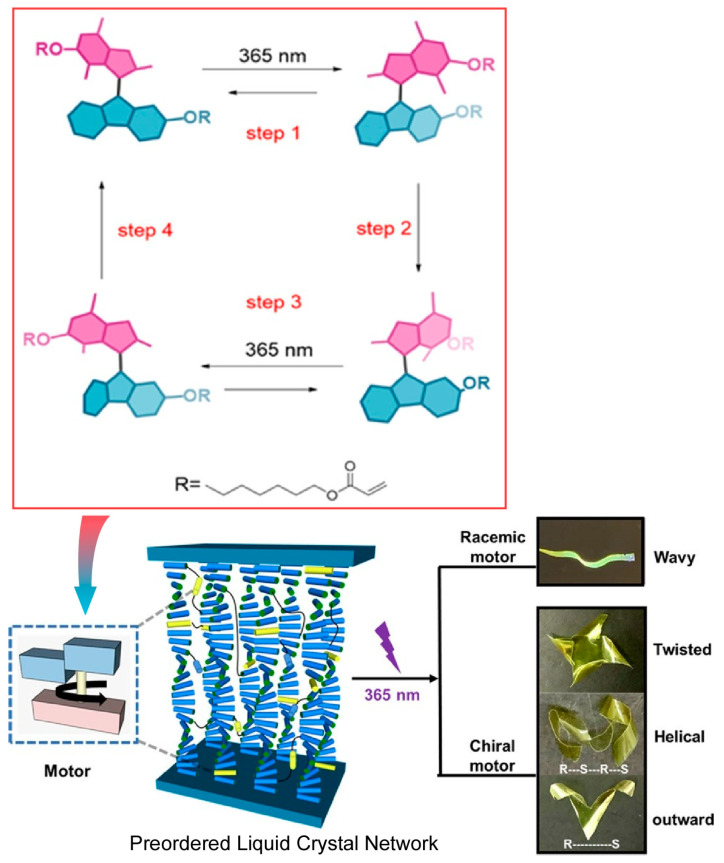
Incorporation of light-driven rotary molecular motors into liquid crystalline polymer net-works to control the dynamic behavior of composite materials. Reproduced under the terms of the CC-BY license [326]. Copyright 2022 American Chemical Society.

Soft and oriented liquid crystal structures provide soft nanospaces. By incorporating molecular functions into the liquid crystal nanospace, macroscopic motion can be induced in the liquid crystal material. Feringa, Chen, and co-workers reported the incorporation of light-driven rotary molecular motors into liquid crystalline polymer networks to control the dynamic behavior of composite materials (Figure 25) [326]. The chiral molecular motors based on sterically dense alkenes used here can be driven by light in a non-invasive manner to rotate in one direction. The rotational cycle process of the motors includes not only molecular geometrical changes but also helicity change steps. It was demonstrated that the motion of the molecular machine due to dynamic chirality was cooperatively amplified, resulting in macroscopic directional motion. First, racemic and enantiomerically pure motors were copolymerized with liquid crystal monomers. In addition, with the help of photolithography technology and other techniques, molecular motors have nanoarchitectonically created a material that is incorporated within an oriented structure in a polymer liquid crystal film. Polymer liquid crystal films containing racemic motors can move on the surface by high-speed wave-like motion, as well as by light-triggered bending. On the other hand, films containing enantiomerically pure motors exhibit synchronized helical motion with different handedness upon UV irradiation. Such studies will explore the possibility of driving photoresponsive materials by programming the rotational motion of molecular motors. The goal will be to design highly responsive and adaptive soft materials and to develop functional materials with complex motility.

Attempts are also being made to drive molecular motors within the nanospace of MOFs. Browne, Feringa, and co-workers have shown that stereoscopically interpenetrated alkene molecular motors can be incorporated into MOF pillars and rotate under visible light [327]. The framework is constructed from two functional sites. Palladium–porphyrin is designed as the linker of the framework and the bispyridyl-derived molecular motor as the pillar. The palladium–porphyrin is a photosensitizer, and the motor portion of the framework is capable of rotational motion using low-energy green light. In other words, this porphyrin skeleton is not only a scaffold, but also absorbs visible light and transfers the collected energy to the molecular motor. This energy transfer process drives the rotational motion of the motor. In fact, an efficient triplet energy transfer between the porphyrin linker and the molecular motor was observed. Due to the good spatial arrangement of the chromophores in the MOF, the energy transfer between the photosensitizer and the molecular motor is efficient. As a result, photochemical isomerization of the molecular motor could be achieved with green 530 nm light. Near-infrared Raman spectroscopy confirms that the visible-light-driven rotation of the molecular motor proceeds in the solid state at a similar rate to that observed in solution. The nanospace of the MOF provides a large free volume. This is an essential nanoarchitectonics element for the unhindered rotation of light-driven molecular motors in the solid state. This study demonstrates that the rotational motion of molecular motors in MOFs can be driven by visible light. It would be possible to apply this technology to molecular membranes and pumps that can accelerate the flow of gases by optical stimulation. Alternatively, miniaturized chemical reactors that can accelerate the inflow of reactants and outflow of products using visible light as a power source could be created in combination with catalytic functions. Furthermore, it may be possible to further shift the excitation wavelength to red light by using photosensitizers or molecular perturbations of molecular motors.

**Figure 26:**
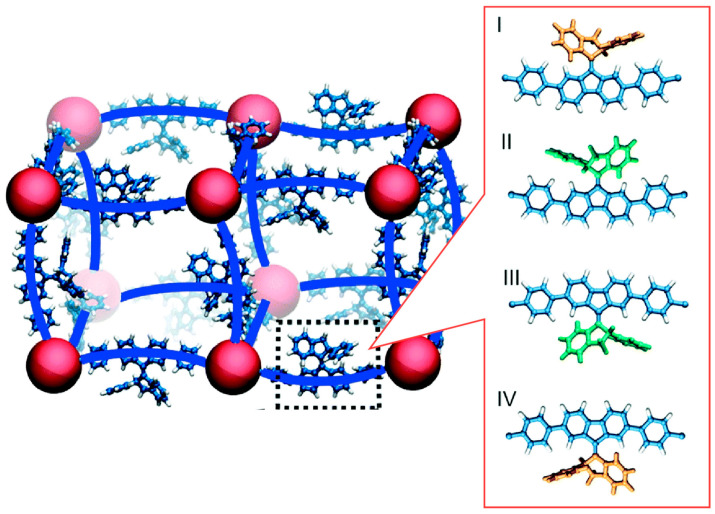
A model for a theoretical approach to elucidate the collective conformational behavior of MOFs incorporating molecular motors. Reprinted with permission from Reference [328]. Copyright 2021 Royal Society of Chemistry.

Theoretical approaches to MOFs incorporating molecular motors have also been made. Photoresponsive molecular motors embedded in the nanospaces of porous materials with some degree of softness, such as MOFs, are expected to behave in a collective and coordinated manner. Such behavior is expected to amplify the motion of individual motor units. However, a thorough understanding of the dominant interactions at the atomic scale of such systems has been lacking. This is a necessary element to predict and fully explore the potential of MOFs incorporating molecular motors. Kolodzeiski and Amirjalayer have advanced a theoretical approach to elucidate the collective conformational behavior of MOFs incorporating molecular motors (Figure 26) [328]. The influence of the interaction between molecular motors on the local and global properties of the framework was pursued by conformational studies. Computational studies were performed for structures with simple cubic MOF-5 topology and organic linkers functionalized by molecular motor units. In particular, the role of linker interactions and the influence of the molecular motor state on the structure of the MOF scaffold were investigated. The symmetry of the motor-functionalized linkers breaks the symmetry of the simple cubic topology. Correspondingly, crystallographic direction-dependent anisotropy arises. These properties are expected to be partially stable at room temperature. It is also revealed that chiral pores and planes can exist within this structure. The latter finding, along with the local chirality of molecular motors, is a promising property for applications such as chiral gas separation, sustainable molecular storage, and sensing. The importance of studying the influence of different network topologies beyond the local structure is demonstrated. A more comprehensive overall picture of the structural properties of molecular motors embedded in highly ordered nanospace structures needs to be revealed. More generally, this will pave the way to elucidate the properties and functions of dynamic properties such as unidirectional rotation as they are reflected at the material level.

**Figure 27:**
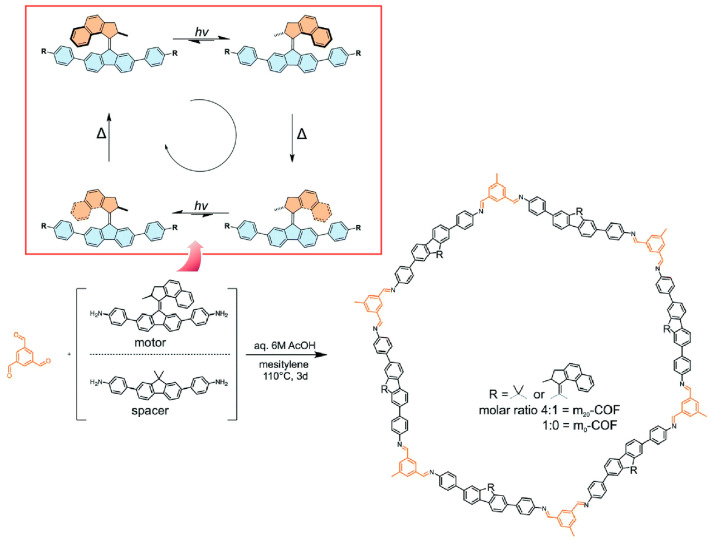
COFs with a diamine-based light-driven molecular motor with the ability to precisely arrange reactive molecules within their crystalline backbone. Reproduced under the terms of the CC-BY license [329]. Copyright 2022 Royal Society of Chemistry.

As seen in the above examples, various functions and phenomena can be studied by incorporating molecular motors into the backbone of porous skeletal structures: for example, non-equilibrium phenomena between the host and guest in three-dimensional solid materials, nanoactuation, and molecular transport. However, the relationship between the structure of the underlying porous nanospaces and the mobility of molecular motors is not fully understood. Lotsch, Feringa, Krause, and co-workers have developed crystallinity-controlled COFs with a diamine-based light-driven molecular motor (Figure 27) [329]. Unlike the use of amorphous polymeric materials or other materials as matrices, COFs, in principle, have the ability to precisely arrange reactive molecules within their crystalline backbone, similar to MOFs. Two-dimensional COF-based nanoarchitectonics can provide a nanospace environment in which both crystallinity and porosity are preserved. A crystalline two-dimensional COF structure with stacked hexagonal layers containing 20 mol% molecular motors was created. The arrangement of molecular motors in the nanospace is crucial for amplifying and coordinating the movement of molecular machines. To this end, the composition, porosity, molecular structure, and skeletal structure of the COFs are investigated. Even if the available pore size exceeds the radius of rotation of the motor, the rotation of the molecular motor is not completely allowed. Intermolecular interactions from adjacent layers may hinder or limit the rotations of the motor. Therefore, it is essential to consider structural properties such as interlayer interactions and stacking offsets resulting from the stacked layers. Using molecular motor units with heteroatom or ^13^C-enriched structures such as fluorine, and advanced in situ solid-state NMR techniques, the rotation of the motors can be observed in detail. The interlayer dynamics and dynamic host-guest properties induced by the light-response dynamics of the molecular motors embedded in the framework can also be studied. Data from such studies can be used for molecular dynamics simulations, providing a multifaceted method to investigate design guidelines for the operation of light-driven molecular motors in porous solids.

Developments in coordination chemistry, supramolecular chemistry, and materials science have made possible the nanoarchitectonics of materials that provide nanospaces that can be precisely designed, as seen in MOFs and COFs. In them, molecular motors and other molecular machines can be immobilized and their movements can be precisely studied. Not only is the correlation between individual molecular motors and space an important target of study, but also the joint movement and accumulation of molecular machines and the interlocking of the movement of molecular machines with matrix materials such as frameworks. The latter two elements, in particular, will also be necessary processes to amplify the nano-level movements of molecular machines to the material level.

## 5. Summary and Perspectives

Thus far, we have been discussing various nanoarchitectonics approaches in different media under various conditions [104,330,331,332]. These previous reviews elucidate the importance of surrounding environments including nanospace confinements. Reevaluations of nanoarchitectonics for dynamic functions and molecular machines on the basis of confined space environments are necessary. Therefore, this review provides an overview of dynamic functions in various confined spaces. They can be summarized as follows. Molecular space is mainly composed of molecules themselves or supramolecular structures. The size of the space is at the molecular level. Therefore, the functions of the molecules and ions trapped there are dynamically perturbed. Such phenomena are useful for elucidating the basic physical properties of functional molecules. The development of coordination chemistry, supramolecular chemistry, and materials science has made it possible to create precisely designed material nanospaces such as MOFs and COFs. Material nanospaces are also associated with large objects and linked to bulk functions. Thus, the functions in materials nanospace can be linked to macroscale phenomena. In addition, the properties of nanospaces in disordered materials remain unexplored and will be the focus of future research. Biomolecules and biostructures have a sophisticated ability to provide nanospace skillfully. By modifying biomolecular systems that originally possess this high capability and expressing functions through confined space, it is possible to modify functions at a higher level. Totally, it can be considered that there is a great potential in the development of functions using bionanospace.

Surface space and internal nanospace are considered as confined spaces for the development of functions of molecular machines. Surface nanospace enables the high-resolution analysis of molecular machines. The details of multi-step and sequential dynamic changes, such as in molecular machines, can be analyzed at high resolution with confinement to the surface. This will reveal more detailed mechanisms of molecular machine operation, paving the way for the advanced analysis of machine behavior and realistic device construction. Molecular motors and other devices can be immobilized in nanospaces within materials such as MOFs and COFs, and their movements can be precisely controlled. In this case, the joint movement and integration of molecular machines and the interlocking of the movement of molecular machines with matrix materials such as frameworks will also be important. This process is also necessary to amplify the nano-functions of molecular machines to the material level.

What these examples show is that not only the central functional unit but also the surrounding spatial configuration is necessary for higher functional expression. In this regard, it can be imagined that the superiorities of bio-systems are due to the fact that biological systems have evolved functional systems based on such design principles. Such a role is played by nanoarchitectonics. It can not only develop functional units but also construct entire systems. For example, external environment-dependent doping can be performed by linking chemical reactions and other processes to the nanospaces that make up organic semiconductors [333]. Such designs can lead to the development of devices and sensors that can respond to the environment [334,335,336]. However, as indicated in this review, confined space is diverse. The number of possible systems is a combination of the diversity of functional units and the diversity of spaces, and thus the number of candidates is enormous. Chemical structures and molecular motions under engineering aspects should be made clearer by summarized discussion on the regarded structures and functions, comparing, for example, length and volume scales, forces, rigidity, stability, robustness time constants, repetition rates, actuation principles, and so on. However, it might be difficult to select the appropriate system from among them only by experimental experience and intuition. Fortunately, mankind has developed artificial intelligence as a means of coping with the enormous information abundance. Methodologies have been proposed for the application of machine learning to chemical/material systems [337,338,339,340,341] and the concept of materials informatics [342,343,344]. For the preparation and use of nanospaces (nanopores, etc.), an attempt to link nanoarchitectonics and materials informatics has also been proposed [345,346]. Functional development is not only about the functional unit itself, but also about the control of the space that accommodates it. Nanoarchitectonics will play important roles in the architecture of such a total system. In doing so, the cooperation of emerging technologies such as artificial intelligence will be necessary.

## Data Availability

Not applicable.
